# Endocarditis following transcatheter or surgical aortic valve replacement: What's the difference?

**DOI:** 10.1002/ccd.30195

**Published:** 2022-04-27

**Authors:** Andrew M. Goldsweig, James B. Hermiller

**Affiliations:** ^1^ Division of Cardiovascular Medicine University of Nebraska Medical Center Omaha Nebraska USA; ^2^ Department of Cardiology Ascension St. Vincent's Heart Center of Indiana Indianapolis Indiana USA

Prosthetic valve endocarditis (PVE) conveys extremely high morbidity and mortality. Accordingly, the American Heart Association (AHA),^1^ the American Association for Thoracic Surgery,^2^ and the European Society of Cardiology^3^ have each issued guidelines for the management of PVE (Table 1). In general, the three societies strongly recommend early surgical intervention for PVE patients with valvular dysfunction, persistent bacteremia, heart block, resistant organisms, recurrent emboli, relapsing infection, and large vegetations. These recommendations, published in 2015 and 2016, predate the widespread utilization of transcatheter aortic valve replacement (TAVR).

**Table 1:** Indications for early surgery for left‐sided prosthetic valve endocarditis (PVE) from the American Heart Association (AHA),^1^ American Association for Thoracic Surgery (AATS),^2^ and European Society of Cardiology (ESC).^3^

Indication	AHA 2015	AATS 2016	ESC 2015
Heart failure from valvular dysfunction	Class I, LoE B	Class I, LoE B	Class I, LoE B
Persistent bacteremia despite appropriate antibiotics	Class I, LoE B	Class I, LoE B	Class IIa, LoE B
Heart block or abscess	Class I, LoE B	Class I, LoE B	Class I, LoE B
Resistant bacteria or any fungi	Class I, LoE B	Class I, LoE B	Class I, LoE C (Class IIa, LoE C for *Staphylococci* or non‐HACEK Gram‐negatives)
Recurrent emboli despite appropriate antibiotics	Class I, LoE B	Class IIa, LoE B	No recommendation
Relapsing PVE	Class I, LoE C	Class IIa, LoE C	No recommendation
Mobile vegetation >10 mm	Class IIb, LoE C	Class IIb, LoE B	Class I, LoE B

Abbreviations: HACEK, *Haemophilus*, *Aggregatibacter*, *Cardiobacterium*, *Eikenella*, *Kingella*; LoE, level of evidence.

In the present issue of this Journal, Bansal et al.^4^ report a retrospective database study of 906 TAVR patients with PVE, of whom 20 (2.21%) underwent surgical aortic valve replacement (SAVR) during the PVE hospitalization. Comparing SAVR to medical therapy for the treatment of post‐TAVR PVE, the authors report no differences in the rates of in‐hospital mortality and 30‐day readmissions, while the SAVR patients had greater lengths of stay and costs of care. In light of these data, should similar PVE management guidelines — recommending early surgical intervention – apply to both TAVR and SAVR valves? We must consider differences in patient populations, valve structure, and PVE outcomes to address this important question.

First, the TAVR and SAVR literatures describe distinctly different patient populations. In the US, TAVR was approved for inoperable and high‐risk patients in 2011, for intermediate‐risk patients in 2016, and for low‐risk patients in 2019. By contrast, SAVR has principally been offered to patients at lower surgical risk. Even today, SAVR is generally reserved for younger patients with prolonged predicted survival per the 2020 AHA/American College of Cardiology guidelines, which give SAVR a class I indication for patients under age 65.^5^ Thus, TAVR patients have, on average, been sicker than their SAVR counterparts, possibly leading to worse PVE surgical outcomes among TAVR patients.

Second, TAVR and SAVR prostheses differ in their structure. TAVR valves' thin‐strut stent frames endothelialize completely, leaving the bioprosthetic leaflets as the only foreign material in contact with the bloodstream. Cardiac output continually washes clean the small neosinuses surrounding TAVR valves. SAVR valves, on the other hand, have thick sewing rings that may never completely endothelialize, and mechanical valves place pyrolytic carbon leaflets into the bloodstream indefinitely. SAVR valves' comparatively large neosinuses have slower flows resulting in greater stasis and risk of thrombus formation. With less exposed prosthetic material and more intrinsic systolic washing, TAVR valves may be easier to sterilize with antibiotics than SAVR valves, possibly leading to better PVE outcomes among TAVR patients treated with medical therapy.

However, outcomes data comparing early surgical versus medical therapy for post‐TAVR and post‐SAVR PVE share many similarities. Bansal et al.^4^ reported no benefit from early surgery in terms of mortality. In the largest prospective study of surgical valve PVE to date, Lalani et al.^6^ found that, after risk adjustment, early reoperation provided no mortality benefit over medical therapy either. Furthermore, the three sets of PVE guidelines that recommend surgery are based entirely upon observation observational data, with no randomized trials comparing surgery to medical therapy for PVE. Indeed, data that early surgery improves outcomes of either post‐TAVR or post‐SAVR PVE more than medical therapy are conspicuously lacking.

Thus, while post‐TAVR and post‐SAVR PVE may differ in their patient populations and valve structures, the exclusively observational data to date provide limited support for early surgery in either valve type. Confirmation bias refers to one's willingness to accept information that supports beliefs one already holds and to reject information that contradicts them. Perhaps the willingness of our professional societies to recommend early surgery for PVE represents a form of communal confirmation bias. Actually, the management of endocarditis following TAVR or SAVR should likely be quite similar: despite strong guideline recommendations for early surgery for PVE, most data show similar outcomes with either surgery or medical management.

## CONFLICTS OF INTEREST

Dr. Andrew M. Goldsweig receives research support from the National Institute of General Medical Sciences, 1U54GM115458, and the UNMC Center for Heart and Vascular Research. Dr. James B. Hermiller Jr. is a consultant and proctor for Edwards Lifesciences and Abbott, and a consultant to Medtronic.
